# Effect of Dynamic Temperature Stimulus to Plantar Surface of the Foot in the Standing Position

**DOI:** 10.3389/fbioe.2016.00088

**Published:** 2016-11-21

**Authors:** Ryo Watanabe, Hiroyuki Kajimoto

**Affiliations:** ^1^Department of Informatics, The University of Electro-Communications, Tokyo, Japan; ^2^JSPS, Tokyo, Japan

**Keywords:** standing posture, postural sway, biomechanics, thermoesthesia, haptic illusion

## Abstract

We have previously found that a vertical force or tactile sensation occurs when the temperature of a participant’s skin changes rapidly. In this illusion, upward motion, pressure, or force sensation is elicited when stimulus temperature rises rapidly, whereas in the opposite case, downward motion or pulling sensation is elicited. In this paper, we applied this phenomenon to the sole (plantar surface of the foot) to present the sensation of ground slope. To investigate this, we conducted an experiment that measured the correlation between stimulation temperature and front–back direction position of the center of gravity. Participants stood on a thermal stimulator on Nintendo Wii Balance Board, and they remained standing during 30-s dynamic temperature stimulus. In result of analysis, it was suggested that dynamic thermal change in sole might influence standing position, and the effect pattern was anomalous in case of the participants who reported a swaying sensation without a haptic sensation. This behavior might be applied to the diagnosis of the presence of thermoesthesia of the patients who might have disease with absence of thermoesthesia.

## Introduction

Relationships have previously been found between thermal sensation and haptic sensations. For example, pressure sensation can be elicited by high temperature water vapor (Kai et al., 2011), and controlling the speed of temperature change at the moment that the fingers touch a surface can provide the illusion of touching different materials (Yamamoto et al., 2006). There is also the example of a somesthetic phenomenon in which a cold object’s weight was felt to be larger than that of an object at a different temperature (Stevens and Green, 1978). Also, it has been suggested that thermal sensation involves the human motor control system. The grip force of participants with congenital insensitivity to pain (CIP), which is defined as an absence of thermal and pain senses (Nagasako et al., 2003), was significantly more volatile and larger than that in normal participants, despite the CIP patients’ other sense and motor functions being normal (Kawashima et al., 2012).

**Figure 1:**
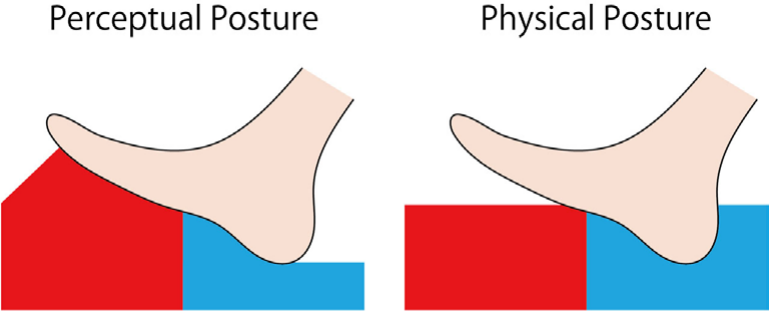
**Expected perception by thermal stimulation to the sole**.

We have found that a force or pressure-like sensation perpendicular to the contact surface is elicited when the temperature of a touched object changes rapidly (Watanabe and Kajimoto, 2014). In this illusion, upward motion, upward pressure, or force sensation is elicited when stimulus temperature rises, whereas in the opposite case, downward motion or pulling sensation is elicited. We speculated that a body sway sensation might be elicited by this thermo-haptic illusion. If an upward sensation arises on one side of the sole of the feet, and a downward sensation arises on the other side following this illusion, the perception of body inclination might be elicited (Figure 1). We also considered that if this swaying sensation could occur, as in our hypothesis, actual body posture might also be influenced by the illusion, as illusory body motion is sometimes associated with real motion, such as when visual flow stimuli generate body sway (Bronstein and Buckwell, 1997), galvanic vestibular stimulation influences walking path (Maeda et al., 2005), and force sensation by skin deformation induces body rotation (Sato et al., 2009). If actual body posture could be controlled by this dynamic temperature stimulus, it could be applied for support system of standing posture.

In this paper, to investigate this hypothesis and its influence on actual posture, we conducted an experiment using a device that can present a dynamic thermal change and measure the position of the center of gravity (COG).

## Materials and Methods

### Experimental System

**Figure 2:**
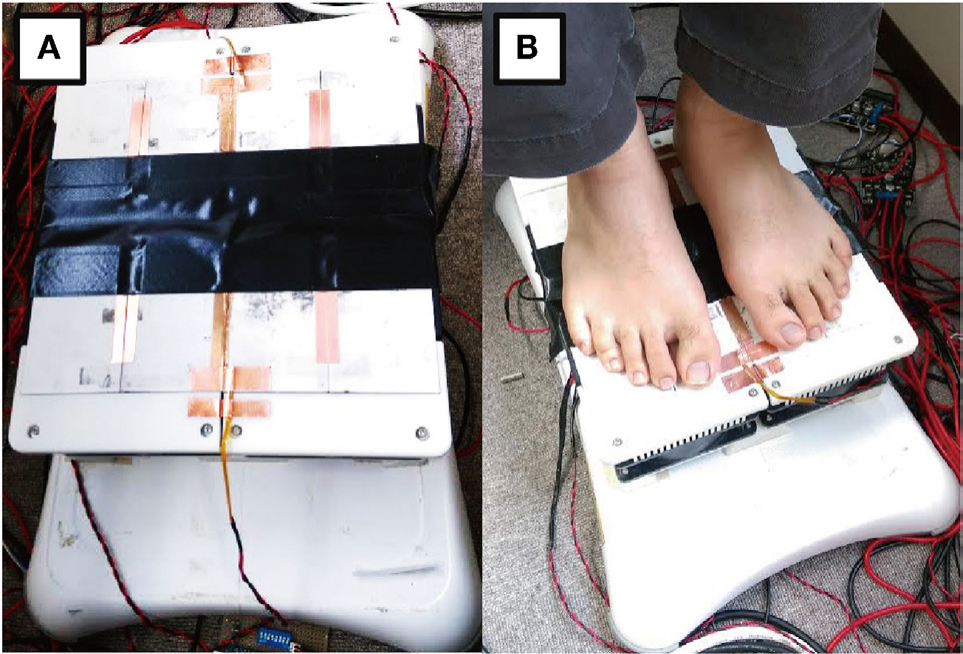
**(A)** Experimental device that presents thermal stimuli to the toe and heel of the sole and tracks the center of gravity. It consists of thermal modules and a stabilometer. **(B)** A participant using the device.

The experimental device consisted of eight Peltier elements (62 mm × 62 mm), heatsinks, fans, thermistors, and a Nintendo Wii Balance Board (WBB) (Nintendo, Japan) (Figure 2). WBB was adopted or following the reason that many previous studies used WBB and suggested that WBB is adaptable and is a low-cost balance testing solution (Clark et al., 2010; Young et al., 2011). The device can present thermal stimuli to the toe and heel part of the sole. The Peltier elements were PI-controlled by a micro controller (mbed NXP LPC1768, NXP, Netherlands) and motor drivers (MDD10A, Cytron Technologies, Malaysia).

### Conditions

Thirty-one (25 male and six female) volunteers aged between 21 and 45 years old participated in this experiment. Table 1 shows demographic characteristics of all participants. Twenty-nine of them participated in bare feet, and two females who participated wore thin stockings. Twenty-four of them had joined previous experiments where dynamic temperature change was stimulated to their sole of feet. The device presented the following five conditions (a–e) of thermal stimuli.

(a) Both the toe and the heel were presented with a dynamic temperature change in the same phase.
(b) Both the toe and the heel were presented with a dynamic temperature change in the opposite phase (i.e., when the toe was heated, the heel was cooled, and *vice versa*).
(c) Only the toes were presented with a dynamic temperature change.
(d) Only the heels were presented with a dynamic temperature change.
(e) Neither the toes nor the heels were presented with a dynamic temperature change.

**Table 1:** **Demographic characteristics of all participants**.

Participant	Sex	Age	Height (cm)	Weight (kg)
1	Female	45	151	48
2	Male	21	159	59
3	Male	31	168	63
4	Male	23	165	76
5	Female	23	172	50
6	Male	28	171	95
7	Male	24	176	76
8	Male	22	174	66
9	Male	25	170	72
10	Male	22	175	53
11	Male	26	171	86
12	Male	23	170	70
13	Male	25	170	50
14	Male	33	158	54
15	Male	21	170	56
16	Male	24	173	58
17	Male	35	175	78
18	Female	24	162	50
19	Male	22	173	64
20	Male	22	164	68
21	Male	26	163	58
22	Female	24	158	48
23	Male	24	164	68
24	Male	24	162	55
25	Male	24	161	47
26	Female	26	166	62
27	Male	40	170	63
28	Male	36	165	63
29	Male	29	174	70
30	Male	23	176	70
31	Female	21	150	51
Average	–	26.3 ± 5.8	167 ± 7	63 ± 12

In conditions (a) through (d), a dynamically changing temperature stimulus following a 0.2-Hz sin wave ranging from 28 to 36°C was presented. These temperatures were selected so as not to damage the skin (ISO13732-1, 2006). The thermal stimuli in these four conditions had the same frequency and the same range, but there were differences in the location and phase of the stimuli. During the experiment, the temperature of the thermal elements was set to 32°C when they did not present a dynamic temperature change.

We expected that participants would feel a haptic sensation in conditions (a) through (d). We also expected that a swaying sensation would be elicited in conditions (b) through (d) by recognizing the difference in level between the toes and the heels (Figure 1).

### Procedure

Participants stood on the device and were asked to remain standing and relax and close their eyes for 30 s. During this 30 s, one of the five stimulus conditions was presented, and the stabilometer traced the transition of the COG. After 30 s, participants were asked to open their eyes, sit down on a chair, and keep their feet on the device. Then they were asked to answer a questionnaire about whether some kind of vertical haptic sensation (moving, tactile, or force) and body swaying sensation were elicited in phase with the temperature change. This questionnaire asked about these sense during temperature stimuli continuance and did not ask about that of after stimuli. All volunteers participated in five trials, one trial per one stimulus condition. The order of trials was randomized. At least 30-s intervals were taken between each trial. All participants did not know the detail of condition, but they only knew that they might be presented dynamic temperature stimuli until all trials finished. This experiment was approved by the University of Electro-Communications Institutional Review Board for Human Subjects Research. All subjects gave written informed consent in accordance with the Declaration of Helsinki.

### Data Analysis

**Figure 3:**
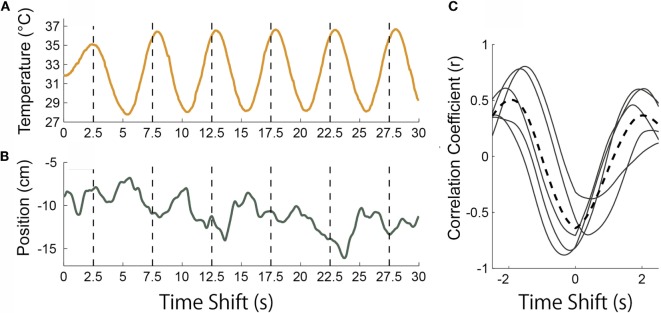
**(A)** An example of stimulus temperature presented to the heel in condition (a). **(B)** Position of center of gravity in the same trial as in **(A)**. **(C)** Results of cross-correlation between temperature and position of center of gravity in increments of every 5 s. The broken curve is the average of these five curves.

To investigate the relationship between the temporal change of the stimulus temperature and that of body sway, the datasets during temperature stimuli continuance were analyzed using cross-correlation methods. Cross-correlation between the temporal change of the stimulus temperature at the heel and that of the position of the COG was calculated for every cycle of temperature change (Figures 3A,B) [in the case of condition (c), in which only the toe was presented with a dynamic temperature change, the toe’s temperature was used for this analysis]. In Figure 3C, solid lines were each five cycle’s correlation curves between temperature and COG, and broken line was the averaged curve of five solid lines. A positive value indicates that participants leaned forward when the temperature to their heels was hot, whereas in the opposite case, they leaned backward when the temperature to their heels was cold [in the case of condition (c), the cross-correlation curves were reversed]. In Figure 3C, this participant’s posture was inclined backward when the temperature increased. The peak point values of these break curves of all trials and participants were used for analysis. These point’s values (correlation values and time shifts) were conducted by multiple Fisher’s LSD test between experimental conditions. Its significant level was 0.05.

## Results

**Figure 4:**
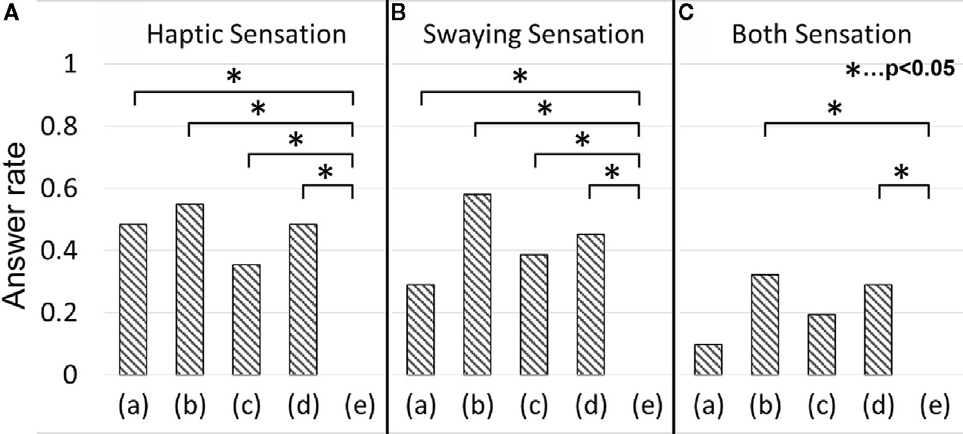
**Answer rate of haptic and swaying sensation**. **(A)** Haptic sensation. **(B)** Swaying sensation. **(C)** Both sensations.

Figure 4 shows the response rates of all 31 participants. Around 50% of all participants reported that they had perceived a haptic sensation in conditions (a) through (d), which generated a dynamic temperature change (Figure 4A). The rate of swaying sensation was about the same for each condition (Figure 4B). The results of a multiple comparison (Steel–Dwass’ test, *p*-values less than 0.05 was considered statistically significant.) showed there were significant differences between condition (e) and the other four conditions in terms of reporting haptic and swaying sensations. Most participants who felt a haptic sensation commented that an upward sensation was elicited when the temperature was rising, whereas in the opposite case, a downward sensation was elicited, which agrees with our previous findings (Watanabe and Kajimoto, 2014). The majority of participants who reported a swaying sensation commented that they felt that their body inclined toward the colder element.

However, contrary to our expectations, in condition (a), a number of participants who reported a swaying sensation were comparable to those who reported a haptic sensation. Participants who felt both haptic and swaying sensations were about half the number of those who felt a swaying sensation (Figure 4C). The results of a multiple Steel–Dwass test showed there were significant differences between conditions (e) and (b), and (e) and (d). In fact, there were certain participants who felt a swaying sensation without a haptic sensation. Some participants reported that it was difficult to perceive the temperature change of their toes. This insufficient toe perception might be because of the small size of the contact area, and this irregularity might cause the swaying sensation reported in condition (a).

**Figure 5:**
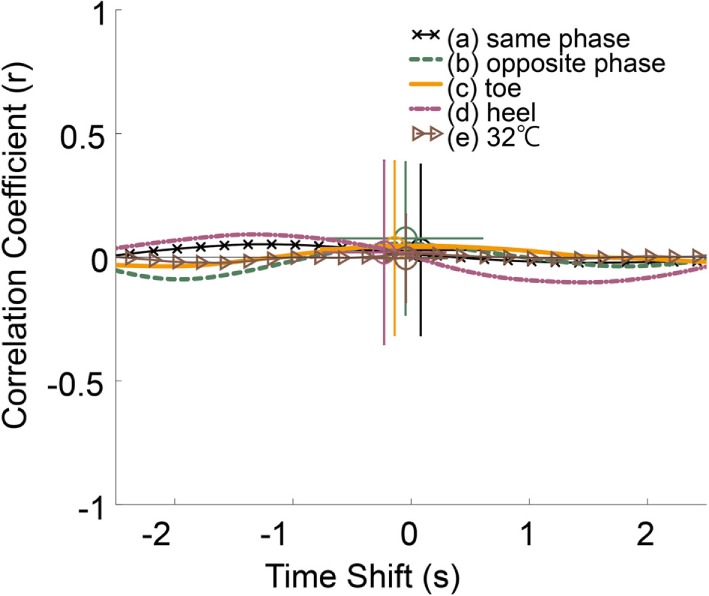
**Averaged cross-correlation curves and averaged peak of cross-correlation of all participants**.

Figure 5 shows the averaged cross-correlation curves between the stimulus temperature and the position of COG for all 31 participants. There were only slight correlations. The results of a multiple Fisher’s LSD test between averaged correlation value and time shifts showed no significant differences. The effect size of correlation value η^2^ was 0.01 and that of time shift was 0.03. The averaged peak values were shown in column “All” in Tables 2 and 3.

**Table 2:** **Correlation values of averaged peak points of each condition and each group**.

Correlation value (*r*)	Mean ± SD				
	All	Haptic	Sway	Only haptic	Only sway
(a) Same phase	0.029 ± 0.345	0.018 ± 0.405	0.169 ± 0.251	−0.059 ± 0.387	0.181 ± 0.283
(b) Opposite phase	0.076 ± 0.309	0.172 ± 0.269	0.084 ± 0.336	0.212 ± 0.214	−0.037 ± 0.354
(c) Toe	0.037 ± 0.352	0.036 ± 0.317	0.165 ± 0.426	0.017 ± 0.215	0.278 ± 0.453
(d) Heel	0.019 ± 0.371	0.128 ± 0.367	−0.054 ± 0.449	0.159 ± 0.264	−0.271 ± 0.437
(e) 32°C	−0.004 ± 0.178	–	–	–	–

**Figure 6:**
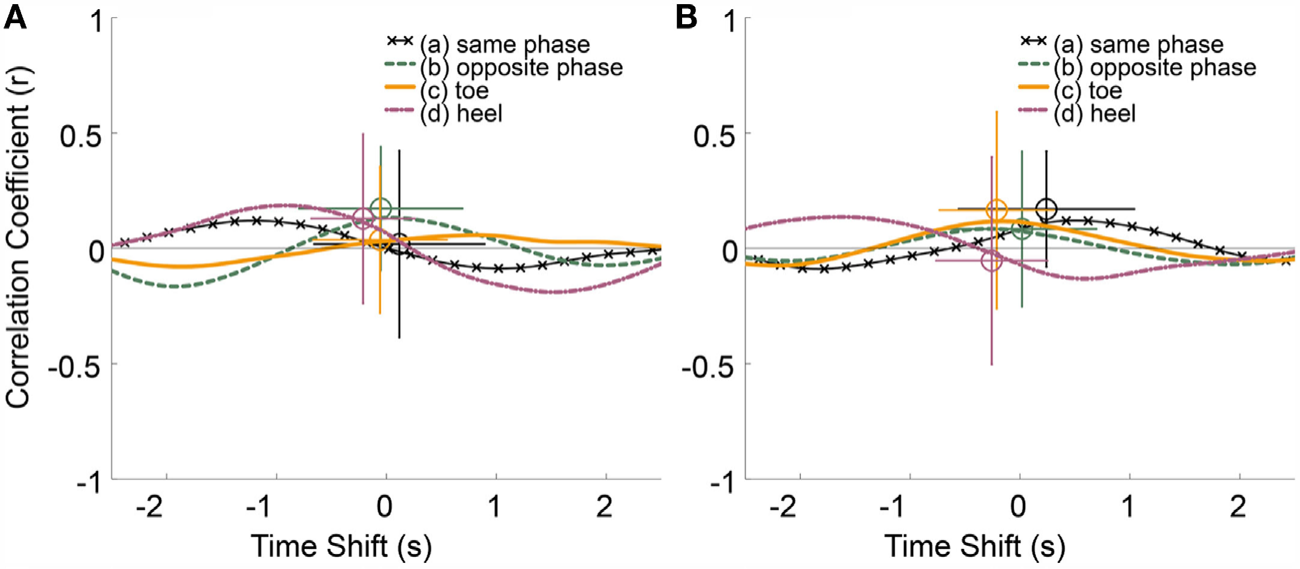
**(A)** Averaged cross-correlation curves and averaged peak of trials in which participants reported a haptic sensation. **(B)** That of trials in which participants reported a swaying sensation.

**Figure 7:**
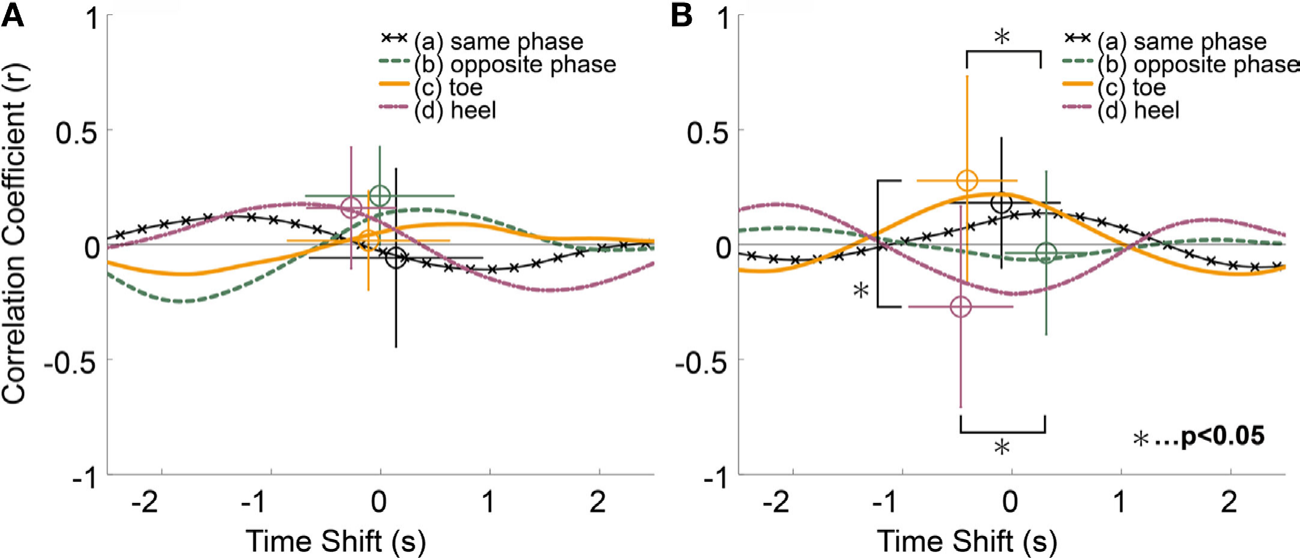
**(A)** Averaged cross-correlation curves and averaged peak of trials in which participants reported only a haptic sensation. **(B)** That of trials in which participants reported only a swaying sensation.

*Group “All” indicates that of all 31 participants; the value that was shown in Figure 5. Groups “Haptic” and “Sway” indicate that of participants who reported haptic or swaying sensation; the values that was shown in Figure 6. Groups “Only haptic” and “Only sway” indicate that of participants who reported only haptic or swaying sensation; the values that were shown in Figure 7*.

**Table 3:** **Time shifts of averaged peak points of each condition and each group**.

Time shift (s)	Mean ± SD				
	All	Haptic	Sway	Only haptic	Only sway
(a) Same phase	0.081 ± 0.626	0.117 ± 0.776	0.240 ± 0.800	0.140 ± 0.789	−0.097 ± 0.535
(b) Opposite phase	−0.047 ± 0.651	−0.052 ± 0.744	0.018 ± 0.678	−0.009 ± 0.675	0.313 ± 0.371
(c) Toe	−0.139 ± 0.624	−0.058 ± 0.609	−0.212 ± 0.522	−0.112 ± 0.739	−0.410 ± 0.453
(d) Heel	−0.228 ± 0.519	−0.213 ± 0.472	−0.257 ± 0.507	−0.270 ± 0.405	−0.468 ± 0.473
(e) 32°C	−0.043 ± 0.349	–	–	–	–

Figure 6 shows the results of the groups of trials in which participants reported haptic (A) or swaying (B) sensations in the questionnaire. These two groups contained common trials. Most of these curves’ shapes indicated that the participants have a few tendencies to lean forward when the temperature to their heels was hot. However, there were opposite tendencies under condition (d) in sway group. The subject size *n* of condition (a) was 15, (b) was 19, (c) was 11, and that of (d) was 14 in haptic group. The *n* of condition (a) was 9, (b) was 18, (c) was 12, and that of (d) was 14 in sway group. We observed a correlation and differing tendencies between these two groups. However, the results of a multiple Fisher’s LSD test showed no significant differences. In haptic group, the effect size of correlation value η^2^ was 0.04 and that of time shift was 0.03. In sway group, the effect size of correlation value η^2^ was 0.06 and that of time shift was 0.08. The averaged peak values were shown in column “Haptic” and “Sway” in Tables 2 and 3.

Figure 7 shows the results of the groups of trials in which participants reported either only haptic (A) or swaying (B) sensations. In this case, these two groups do not contain common trials. Most of these curves’ shapes indicated that the participants have a tendency to lean forward when the temperature to their heels was hot. However, there was opposite tendency under condition (d) in sway group. The subject size *n* of condition (a) was 12, (b) was 7, (c) was 5, and that of (d) was 6 in haptic group. The *n* of condition (a) was six, (b) was eight, (c) was six, and that of (d) was five in sway group. The results of a multivariable Fisher’s LSD test showed that there were three significant differences in only swaying group (Figure 7B). One of that was between conditions (c) and (d) of correlation value. Other two of that were between conditions (b) and (c), (b) and (d) of time shift. In only haptic group, the effect size of correlation value η^2^ was 0.14 and that of time shift was 0.06. In only sway group, the effect size of correlation value η^2^ was 0.24 and that of time shift was 0.38. The averaged peak values were shown in column “Only haptic” and “Only sway” in Tables 2 and 3.

## Discussion

### Causes of Swaying Sensation

We expected that a swaying sensation would be elicited when participants perceived two vertical opposite haptic sensations. However, there were some trials in which participants felt a swaying sensation without a haptic sensation, the amount of which was comparable to that of the trials with both haptic and swaying sensations. Therefore, this swaying sensation might have roots in a cause other than the misconception of ground inclination by the thermos-haptic illusion. We inferred the causes of this swaying sensation from the fact that most participants responded that they felt their body inclined toward cold elements when a dynamic temperature change was presented. This perspective is associated with a thermal phenomenon called the Weber phenomenon. In 1846, E. H. Weber observed that a cold coin resting on the forehead was felt to be heavier than a warm coin. This somesthetic phenomenon was also observed in the whole body (palm, forearm, abdomen, thigh, upper arm, and back) (Stevens and Green, 1978; Stevens, 1979). If this phenomenon could also be generated at the sole, it is conceivable that participants felt that some parts of their sole on a cold area became heavier than on another area and construe this illusory weight growth as moving COG; in other words, an inclination of their body.

### Difference of Response to Thermal Stimulus between Participants’ Answers

There seemed to be different tendencies between participants’ perceptions with regard to haptic and swaying sensations. In the case of the “Only haptic” group (Figure 7A), there were positive peaks in conditions (a), (b), and (d), which represented dynamic temperature change to the heels. In case of the “Only swaying” group (Figure 7B), there were positive peaks in conditions (a) and (c), a negative peak in condition (d), and a flat curve in condition (b). This result indicates that there are opposite natures between the toe and the heel for trials in which participants felt only a swaying sensation. The toes tried to avoid a hot floor and cling to a cold floor, whereas the heels tried to cling to hot and avoid cold in the “Only swaying” group. From this perspective, the behavior in conditions (a) and (b) of this group becomes more understandable. In condition (a), the toe and heel were presented with the same temperature change and produced opposite behavior. In condition (b), the toe and heel were presented with the opposite phase temperature change and, as a result, the toe and the heel produced the same behavior, canceling each other out. There were also differences about time lags between the groups. There was larger dispersion of time lags of peaks between each stimuli conditions in “Only swaying” group than that of “Only haptic” group.

### Implications and Limitations

Before this experiment, it was assumed that if dynamic temperature stimuli to sole could control gravity center position of most people, it could be applied to support or control system of standing posture. However, the result indicates that there were different actual postural sways by participants’ perception. Therefore, it is difficult that this behavior will be applied to support or control system for all people. We thought that this behavior might be applied to one of the diagnosis method of thermoesthesia detection. It might be particularly useful in detection of the diseases with absence of thermoesthesia (e.g., diabetic or CIP patient’s).

The effect that was investigated in this paper was limited during just temperature stimuli continuance. Investigation of the persistence of this effect is one of the important future works.

## Conclusion

We proposed eliciting a body swaying sensation using a phenomenon in which force sensation is associated with dynamic temperature change. We conducted an experiment, obtained subjective impressions, and measured the correlation between stimulus temperature changes and front–back sway of the COG. About half of the participants reported a haptic or swaying sensation, as we expected. However, contrary to our expectation, there were certain participants who felt a swaying sensation without a haptic sensation or a swaying sensation when both toes and heels were stimulated at the same phase. The physical behavior of trials in which participants felt a haptic sensation agreed with our expectations. However, the behavior of trials in which participants felt only a swaying sensation in the heel stimulation condition differed from our expectations.

## Author Contributions

RW designed the study, collected, and analyzed the data and also wrote the initial draft of the manuscript. HK critically reviewed and assisted in the preparation of the manuscript. The final version of the manuscript was approved by both the authors.

## Conflict of Interest Statement

The authors declare that the research was conducted in the absence of any commercial or financial relationships that could be construed as a potential conflict of interest.
